# Antioxidant and Anti-Inflammatory Effects of Crude *Gastrodia elata* Polysaccharides in UVB-Induced Acute Skin Damage

**DOI:** 10.3390/antiox14070894

**Published:** 2025-07-21

**Authors:** Jiajia Liu, Xiaoqi Yang, Xing Huang, Yuan Luo, Qilin Zhang, Feng Wang, Yicen Lin, Lianbing Lin

**Affiliations:** 1 Faculty of Life Science and Technology, Kunming University of Science and Technology, Kunming 650500, China; liujiajialjj@163.com (J.L.); yangxiaoqi991@163.com (X.Y.); hx0802315@163.com (X.H.); lyuany202507@163.com (Y.L.); zhangql@kust.edu.cn (Q.Z.); wangf@kust.edu.cn (F.W.); 2 Engineering Research Center for Replacement Technology of Feed Antibiotics of Yunnan College, Kunming 650500, China

**Keywords:** ultraviolet radiation B, crude *Gastrodia elata* polysaccharides, antioxidant, anti-inflammatory, skin repair

## Abstract

Ultraviolet B (UVB) irradiation drives skin photodamage, prompting exploration of natural therapeutics. This study investigated the reparative effects and mechanisms of crude *Gastrodia elata* polysaccharides (GP) on UVB-induced acute skin damage. GP was extracted from fresh *G. elata* via water extraction and alcohol precipitation. It is a homogeneous polysaccharide with a weight-average molecular weight of 808.863 kDa, comprising *Ara*, *Glc*, *Fru*, and *GalA*. Histopathological analysis revealed that topical application of GP on the dorsal skin of mice effectively restored normal physiological structure, suppressing epidermal hyperplasia and collagen degradation. Biochemical assays showed that GP significantly reduced the activities of MPO and MDA following UVB exposure while restoring the enzymatic activities of SOD and GSH, thereby mitigating oxidative stress. Moreover, GP treatment markedly upregulated the anti-inflammatory cytokines TGF-β and IL-10 and downregulated the pro-inflammatory mediators IL-6, IL-1β, and TNF-α, suggesting robust anti-inflammatory effects. Transcriptomics revealed dual-phase mechanisms: Early repair (day 5) involved GP-mediated suppression of hyper inflammation and accelerated necrotic tissue clearance via pathway network modulation. Late phase (day 18) featured enhanced anti-inflammatory, antioxidant, and tissue regeneration processes through energy-sufficient, low-inflammatory pathway networks. Through a synergistic response involving antioxidation, anti-inflammation, promotion of collagen synthesis, and acceleration of skin barrier repair, GP achieves comprehensive repair of UVB-induced acute skin damage. Our findings not only establish GP as a potent natural alternative to synthetic photoprotective agents but also reveal novel pathway network interactions governing polysaccharide-mediated skin regeneration.

## 1. Introduction

As the interface between the body and the external environment, the skin serves as the primary barrier against external stressors [1]. Excessive exposure to ultraviolet (UV) radiation can lead to skin damage, erythema, melanin deposition, and even cancer [2]. Among UV rays, UVB is the most destructive to the skin, with a harm potency 1000-fold greater than UVA at equivalent dose [3]. Overexposure to UVB triggers synergistic pathways, including oxidative stress, DNA damage, inflammatory cytokine release, collagen degradation, and apoptosis, ultimately resulting in sunburn, inflammation, photoaging, and carcinogenesis [4,5,6].

Under excessive UVB irradiation, the skin generates a surge of reactive oxygen species (ROS), inducing oxidative stress. Under normal physiological conditions, ROS can be neutralized by antioxidant enzymes such as superoxide dismutase (SOD) and glutathione peroxidase (GSH-Px) [7]. However, ROS overproduction depletes antioxidant enzymes, compromising systemic antioxidant capacity and leading to oxidative cellular damage and apoptosis [8]. Additionally, UVB overexposure stimulates pro-inflammatory cytokine release, such as interleukin-6 (IL-6), interleukin-1β (IL-1β), and tumor necrosis factor-α (TNF-α), thereby initiating an inflammatory response [9]. These cytokines further stimulate ROS production, creating a vicious cycle that exacerbates skin tissue damage, collagen degradation, and photoaging [10].

After UVB exposure, the skin initiates self-repair mechanisms, including epidermal thickening, DNA repair, and antioxidant system activation [11]. However, when UVB exceeds intrinsic repair capacity, sunscreens and antioxidants are required to alleviate acute photodamage [12]. Conventional chemical sunscreens, such as UV absorbers and blockers, may provoke inflammation, allergies, and other side effects [13]. Common antioxidant additives, including vitamin C (VC), vitamin E (VE), idebenone (IDE) [14], and chlorogenic acid (CGA) [15], have been widely used. However, long-term, high-dose usage of unstable, low-activity antioxidants may cause side effects such as diarrhea and gastrointestinal disorders [16]. Therefore, developing natural compounds with antioxidant and anti-inflammatory properties is crucial. Polysaccharides have been shown to exert photoprotective effects against UVB-induced skin damage [17,18,19].

The molecular mechanisms of polysaccharides in treating skin photodamage mainly focus on regulating UV-induced signaling pathways. *Dendrobium nobile* Lindl. polysaccharides improve UVB-induced oxidative damage and apoptosis in HaCaT cells by modulating the MAPKs signaling pathway [20]. Downregulated JAK-STAT signaling pathway plays a role in *Agaricus blazei* Murill polysaccharide-mediated photoprotection [21]. Similarly, the *Lycium barbarum* polysaccharide showed partial protection potential against UVB irradiation-induced photo-damage by activation of the Nrf2/ARE pathway, and also suppressed the UVB-induced p38 MAPK pathway [22]. However, most studies focus on isolated pathways, with limited exploration of crosstalk or hierarchical interactions between pathway networks.

*Gastrodia elata* Bl., an edible and medicinal plant, exhibits neuroprotective, antioxidant, anti-inflammatory, and cardioprotective properties [23]. It is widely used in the cultivation and processing of traditional Chinese medicine, the development of related food and health products, as well as skincare products, resulting in an enormous market demand. Polysaccharides constitute the most abundant bioactive fraction of *G. elata* [24]. Due to their high safety profile and therapeutic potential, *G. elata* polysaccharides have received increasing attention for diverse biological activities. However, current studies predominantly focus on their anticancer, immunomodulatory, and neuroprotective effects [25,26,27,28], with limited investigation into their applicability for treating cutaneous photodamage. The underlying mechanisms remain largely unexplored. To determine whether crude *G. elata* polysaccharide (GP) could serve as an effective natural alternative to synthetic photoprotectants and elucidate its reparative mechanisms against UVB-induced acute skin damage, we first characterized the molecular properties of GP. We then evaluated its therapeutic efficacy in a UVB-induced acute skin damage model. Comprehensive histopathological, biochemical, and transcriptomic analyses were integrated to decipher its repair mechanisms.

## 2. Materials and Methods

### 2.1. Extraction of GP

Fresh *G. elata* rhizomes were harvested in 2024 from a cultivation base in Xiaocaoba Town, Zhaotong City, Yunnan Province (27.78° N, 104.60° E). Fresh rhizomes were homogenized with sterile water at a ratio of 1:4 (*w*/*v*) to produce *G. elata* homogenate. A portion of the homogenate was reserved for subsequent experiments, while the remainder was processed for polysaccharide extraction. Four times the volume of sterile water was added to the homogenate, followed by sonication for 20 min and incubation in a 75 °C water bath for 80 min. The crude sugar solution was obtained by centrifugation at 9000 rpm for 10 min. The supernatant was concentrated to 1/3 of its original volume at 50 °C using a rotary evaporator. Proteins were removed using the Sevag method, in which the Sevag reagent (chloroform: n-butanol = 4:1) was added to the crude polysaccharide solution at a ratio of 1:5 (reagent to solution). Subsequently, the deproteinized crude polysaccharide solution was precipitated overnight at 4 °C with four volumes of anhydrous ethanol. The precipitate was collected, dissolved in distilled water, filtered to remove insoluble residues, and lyophilized to yield GP powder.

### 2.2. Molecular Weight Determination

The molecular weight of GP was analyzed using a ThermoFisher ICS 5000 high-performance gel permeation chromatography (HPGPC) system (Waltham, MA, USA). A refractive index detector (RID-20A) was used to measure the retention time of the sample. Separation was performed on a BRT105-103-101 column (8 × 300 mm) with 0.05 M NaCl as the mobile phase at a flow rate of 0.7 mL/min. A dextran standard series was used to calibrate the linear regression curve (*y* = −0.1179*x* + 9.1556, *R*^2^ = 0.9947), from which the molecular weight of GP was calculated.

### 2.3. Monosaccharide Composition Analysis

The monosaccharide composition of GP was analyzed using a high-performance liquid chromatography system (HPLC) (Thermo Fisher Scientific, Waltham, MA, USA). Briefly, 5 mg of GP was hydrolyzed with 3 M trifluoroacetic acid (TFA) at 120 °C for 3 h. After cooling to room temperature, the solvent was evaporated under nitrogen at 40 °C. The residue was reconstituted in 5 mL water, vortexed, and centrifuged at 12,000 rpm for 5 min. The supernatant was analyzed on a Dionex Carbopac™ PA10 column (4 × 250 mm) with mobile phases A (H_2_O), B (500 mM NaOH + 50 mM NaOAc), and C (20 mM NaOH) at a flow rate of 1.0 mL/min (30 °C column temperature). The gradient program was: 0–30 min, 0–50% A; 30.1–46 min, 70% A. Injection volume: 25 µL.

### 2.4. Fourier-Transform Infrared (FT-IR) Spectroscopy

Dried GP (2 mg) was mixed with 200 mg anhydrous KBr, ground, and pressed into a pellet. A blank control pellet was prepared from pure KBr powder. Both pellets were analyzed using a Fourier transform infrared spectrometer (FT-IR 650, Tianjin Gangdong Sci. & Tech. Development Co., Ltd., Tianjin, China) with spectra acquired over 32 co-added scans across 4000–400 cm^−1^ at 0.5 cm^−1^ resolution. Spectral data processing—including absorbance/transmittance conversion and automated smoothing—was performed using OMNIC software (v9.2, Thermo Nicolet Corporation Inc., Madison, WI, USA).

### 2.5. Scanning Electron Microscopy (SEM)

The morphological features of GP were characterized using an ultra-high-resolution scanning electron microscope (SU8600, Hitachi, Chiyoda, Japan) equipped with secondary electron detectors (UD and LD, Hitachi, Japan). Lyophilized GP (5 mg) was adhered to a conductive carbon tape, sputter-coated with gold for 40 s, and imaged using a scanning electron microscope at 5 kV accelerating voltage.

### 2.6. Animal Experiments

Five-week-old male C57BL/6J mice were purchased from SPF (Beijing) Biotechnology Co., Ltd. After one week of acclimatization, mice were housed under standard conditions (22 ± 2 °C, 50–60% humidity, 12/12 h light/dark cycle) with free access to food and water. All protocols were approved by the Animal Welfare and Ethics Committee of Kunming University of Science and Technology (PZWH (Dian) K2023-0007).

**Figure 1:**
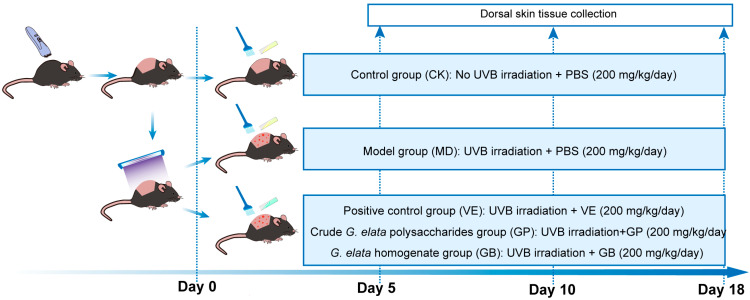
The timeline of model establishment, treatment, and sample collection in mice.

A UVB-induced acute skin damage model was established by incremental UVB exposure through an ultraviolet mutagenesis chamber (Model ZW-15, Shaoxing Zhicheng Instrument Co., Ltd., Zhejiang, China). Mice were anesthetized, depilated, and irradiated in a UV chamber at a distance of 15 cm from UVB lamps (two 15 W UVB lamps, λmax = 313 nm, irradiance 1.5 mW/cm^2^). The exposure lasted for three days, with durations of 50 s, 50 s, and 43.3 s, and a total exposure dose not exceeding 430 mJ/cm^2^ (Dose (mJ/cm^2^) = Intensity (mW/cm^2^) × Time (s)) [29,30]. Mice were randomized into five groups (*n* = 9/group) with the following treatments (Figure 1): control group (CK) without UVB exposure, treated with PBS (200 mg/kg/d); model group (MD) exposed to UVB, treated with PBS (200 mg/kg/d); positive control group (VE) exposed to UVB, treated with VE (200 mg/kg/d); *G. elata* homogenate group (GB) exposed to UVB, treated with *G. elata* homogenate (200 mg/kg/d); GP group exposed to UVB, treated with GP (200 mg/kg/d). Three mice per group were sacrificed on days 5, 10, and 18 for dorsal skin tissue collection (Figure 1). The samples were immediately flash-frozen in liquid nitrogen and stored at −80 °C for subsequent analysis.

### 2.7. Skin Phenotype Evaluation

Daily macroscopic changes were scored by 15 researchers based on the scoring standard of disease activity index (DAI) (Table S1) [31]. Additionally, daily photographs of the dorsal skin were taken before drug administration. Skin samples were fixed in 4% paraformaldehyde, paraffin-embedded, sectioned, and stained with hematoxylin and eosin (H&E) or Masson’s trichrome for histological analysis via light microscopy.

### 2.8. Antioxidant Profile Analysis

Dorsal skin tissues were weighed, homogenized in PBS, and centrifuged (3000 rpm, 20 min). Supernatants were aliquoted and stored at −80 °C until analysis. Levels of malondialdehyde (MDA), myeloperoxidase (MPO), GSH, and SOD in supernatants were quantified using enzyme-linked immunosorbent assay (ELISA) kits (Saibai Biotechnology Co., Ltd., Wuhan, China).

### 2.9. Inflammatory Cytokine Assay

Pro-inflammatory cytokines IL-6, IL-1β, and TNF-α and anti-inflammatory cytokines transforming growth factor-β (TGF-β) and interleukin-10 (IL-10) in supernatants were measured using ELISA kits (spbio Biotechnology Co., Ltd., Wuhan, China).

### 2.10. Transcriptomic Sequencing and Analysis

Transcriptome sequencing was performed on dorsal skin tissue samples collected on days 5 and 18. Total RNA was isolated using the Trizol Reagent (Invitrogen Life Technologies), after which the concentration, quality, and integrity were determined using a NanoDrop spectrophotometer (Thermo Scientific). Oligo-dT was used to enrich mRNA, which was fragmented and synthesized into cDNA for library construction. Next-generation sequencing (NGS) was performed using the Illumina sequencing platform. Pearson correlation coefficients represented gene expression levels between samples. Differential gene expression was analyzed using DESeq2 (v1.38.3) under the conditions |log2FoldChange| > 1 and *p*-value < 0.05. Network enrichment analysis was completed using the Majorbio cloud platform and visualized with Cytoscape (v3.3.0). Other analyses, including principal component analysis (PCA), volcano plot, Venn diagram, UpSet plot, KEGG enrichment analysis, were performed using the free online platform Personalbio GenesCloud.

### 2.11. Data Analysis

Data were analyzed using SPSS (v23.0, IBM, New York, NY, USA). Normality was assessed by Shapiro–Wilk tests. Homogeneity of variance was confirmed via Levene’s test. One-way ANOVA with Tukey’s post hoc test was applied for multi-group comparisons. Significance threshold: *p* < 0.05.

## 3. Results

### 3.1. Molecular Weight, Monosaccharide Composition, and Structural Analysis of GP

**Figure 2:**
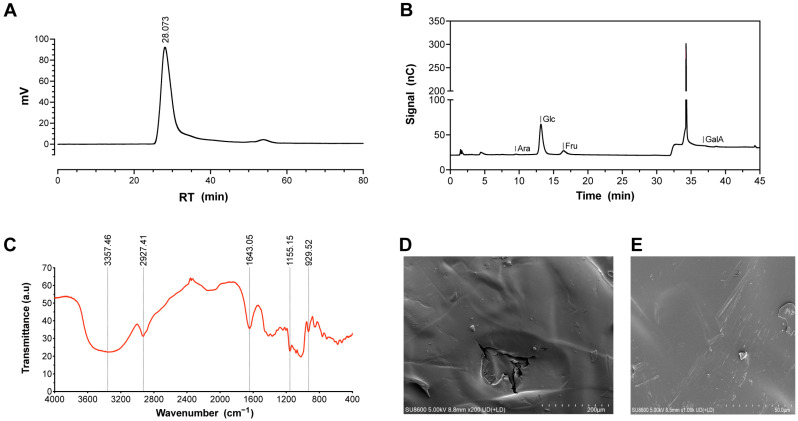
Structural characteristics of GP. (**A**) The result of the weight-average molecular weight of GP. (**B**) The result of monosaccharide composition of GP. (**C**) The FT-IR spectrum of GP. (**D**) SEM image (200×) of GP. (**E**) SEM image (1000×) of GP.

The molecular weight and purity of GP were determined by HPGPC. The chromatogram displayed a single symmetrical peak (Figure 2A), indicating a relatively homogeneous polysaccharide. The weight-average molecular weight (Mw) was calculated as 808.863 kDa (*LgMw* = −0.1113x + 9.0324, *R*^2^ = 0.9944). HPLC analysis (Figure 2B) revealed four monosaccharide peaks corresponding to *Arabinose* (*Ara*), *Glucose* (*Glc*), *Fructose* (*Fru*), and *Galacturonic acid* (*GalA*) in a molar ratio of 1.13:66.12:31.76:0.99. Trace *GalA* suggested weak acidity in GP.

FT-IR spectroscopy (Figure 2C) revealed a broad absorption band in the range of 3600–3200 cm^−1^, corresponding to the stretching vibrations of hydroxyl (-OH) groups, which are characteristic of polysaccharides. Strong absorption peaks were detected at 3357 cm^−1^, 2927 cm^−1^, 1643 cm^−1^, and 1155 cm^−1^, corresponding to the stretching vibrations of O-H, C-H, C=O, and C-O bonds, respectively, indicating the presence of glycosidic linkages in GP. Absorption peaks at 1238 cm^−1^, 1203 cm^−1^, and 1020 cm^−1^ were attributed to O-H bending vibrations, further confirming the presence of hydroxyl groups and the formation of a hydrogen bonding network. An absorption peak at 929 cm^−1^ was assigned to the asymmetric ring structure of pyranose, suggesting that GP consists of sugar units predominantly in the six-membered pyranose form. Additionally, a peak at 852 cm^−1^ was associated with the bending vibration of the C-H bond in α-anomers, indicating the presence of α-glycosidic linkages in GP. SEM imaging (Figure 2D,E) showed smooth, flaky GP particles with minor surface irregularities at 200× magnification and microscopic scratches/protrusions at 1000×.

### 3.2. GP Repairs UVB-Induced Acute Skin Damage in Mice

**Figure 3:**
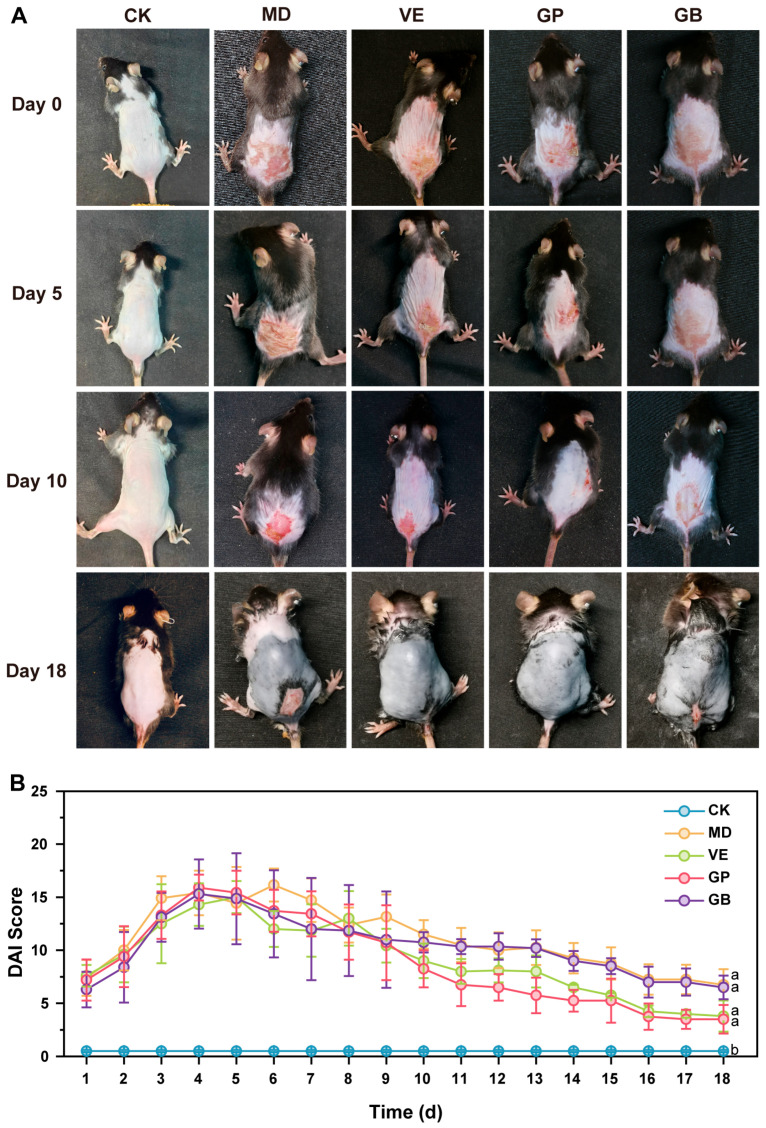
In vivo skin condition of mice. (**A**) The appearance of mice skin was observed via the naked eye on days 0, 5, 10, and 18. (**B**) DAI score of the dorsal skin in mice. Different letters indicate statistically significant differences (*p* < 0.05).

Macroscopic evaluation (Figure 3A) revealed acute photodamage characteristics, including skin thickening, wrinkling, erythema, edema, and necrosis in UVB-exposed groups (MD, VE, GP, GB) by day 0 (after UVB irradiation). The severity of skin damage peaked between days 4 and 6, as indicated by the highest DAI scores (Figure 3B). By day 10, the GP group exhibited superior recovery (DAI score: 8.25 ± 1.75) compared to VE (9.00 ± 1.62), GB (10.75 ± 0.96), and MD (11.50 ± 1.35). By Day 18, GP (3.50 ± 1.35) and VE (3.80 ± 1.47) groups showed near-complete resolution, while GB (6.50 ± 1.12) and MD (6.75 ± 1.47) remained significantly impaired. Over time, the skin condition in all groups gradually improved, with a progressive decrease in DAI scores. Both GP and VE significantly repaired UVB-induced epidermal damage.

**Figure 4:**
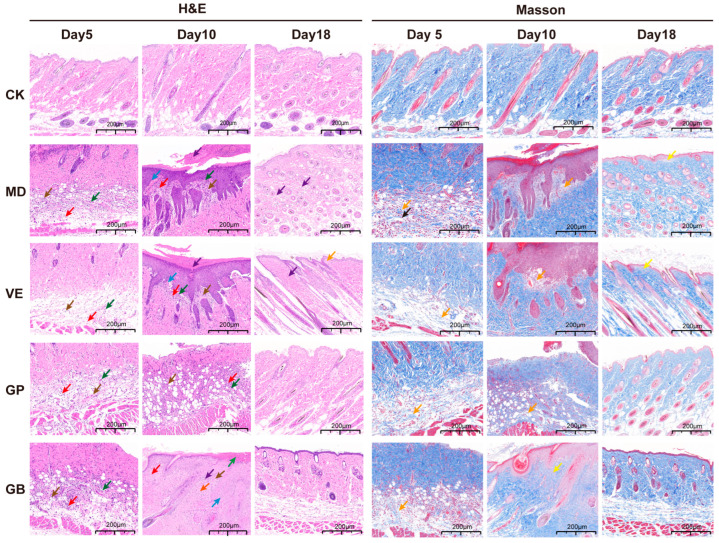
Histopathological analysis of the dorsal skin. Black arrow: necrosis; Purple arrow: parakeratosis; Brown arrow: hyperkeratosis; Green arrow: neovascularization; Red arrow: lymphocytic and granulocytic infiltration; Orange arrow: loosely arranged and irregular collagen fibers; Blue arrow: acanthosis (epidermal hyperplasia); Yellow arrow: collagen hyperplasia.

H&E staining (Figure 4) revealed that on day 5, compared to the CK group, the other four groups exhibited varying degrees of epidermal damage, including structural disorganization, indistinct boundaries, necrosis, and a substantial infiltration of lymphocytes and granulocytes. By day 10, the epidermis of the GP, VE, and GB groups became more intact, with well-defined layer structures, although some necrotic areas and minor lymphocyte and granulocyte infiltration persisted in the dermis and subcutaneous tissue. The MD group displayed hyperkeratosis, parakeratosis, localized epidermal thickening, mild basal cell proliferation, minor lymphocyte and granulocyte infiltration, and slight hemorrhage. By Day 18, the GP and VE groups exhibited complete and relatively thin epidermis, with neatly arranged collagen fibers in the dermis, well-distributed skin appendages (hair follicles and sebaceous glands), and minimal lymphocyte infiltration. In the GB group, a slight increase in fibrous connective tissue proliferation was observed in the superficial dermis, with mild granulocyte and lymphocyte infiltration in the dermis and subcutaneous tissue. The MD group still showed areas of parakeratosis, uneven epidermal thickness, minor lymphocyte infiltration, neovascular congestion, slight hemorrhage, and a reduced number of skin appendages, with some follicles containing keratin plugs.

Masson’s trichrome staining (Figure 4) confirmed collagen degradation in four UVB groups at day 5. By Day 18, the GP group exhibited abundant collagen fibers in the dermis without significant proliferation, whereas the MD, VE, and GB groups still displayed varying degrees of collagen fiber reduction or proliferation. Overall, with increasing treatment duration, skin tissue gradually recovered in all groups. GP, VE, and GB were all effective in repairing UVB-induced skin tissue damage, with GP demonstrating the most significant effect, followed by VE.

### 3.3. GP Modulates Oxidative Stress Biomarkers

**Figure 5:**
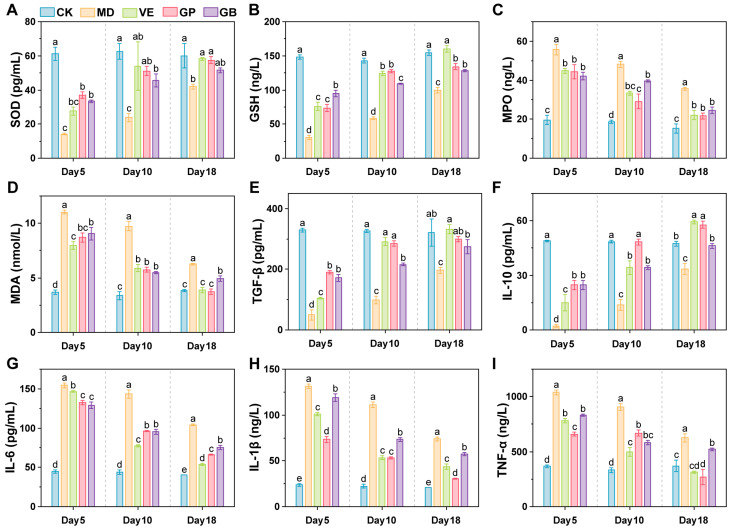
Effects of GP intervention on various biochemical indexes in the dorsal skin of mice. Expression of SOD (**A**), GSH (**B**), MPO (**C**), MDA (**D**), TGF-β (**E**), IL-10 (**F**), IL-6 (**G**), IL-1β (**H**), TNF-α (**I**). Different lower letters indicate significant differences among the groups (*p* < 0.05).

UVB-exposed groups showed increasing GSH and SOD and decreasing MDA and MPO levels over time (Figure 5A–D). The MD group exhibited significantly lower GSH and SOD (*p*-values shown Table S2) and higher MDA and MPO (*p* < 0.001) than other groups. On day 5, the SOD level in the GP group was slightly higher than that in the GB and VE groups. By day 10, GP significantly outperformed GB in elevating GSH (*p* < 0.001) and reducing MPO (*p* < 0.01). By Day 18, the GP group exhibited slightly higher SOD and GSH levels compared to the GB group, whereas MDA levels were significantly lower (*p* < 0.01). Overall, GP, GB, and VE enhanced GSH and SOD levels while reducing MDA and MPO levels in UVB-damaged skin tissue, with GP showing superior efficacy to GB and comparable effects to VE.

### 3.4. GP Regulates Inflammatory Cytokines

Pro-inflammatory cytokines (IL-6, IL-1β, TNF-α) decreased, while anti-inflammatory factors (TGF-β, IL-10) increased in UVB-exposed groups (Figure 5E–I). In mice in the UVB-exposed groups, TGF-β and IL-10 levels increased over time, while IL-6, IL-1β, and TNF-α levels decreased. The MD group exhibited significantly lower TGF-β and IL-10 levels (*p*-values in the Table S3) and higher IL-6, IL-1β, and TNF-α levels compared to the other four groups. On Day 5, the GP group showed significantly higher TGF-β and IL-10 levels (*p* < 0.001) than the VE group, while IL-6, IL-1β, and TNF-α levels were significantly lower (*p* < 0.001) than those in the VE and GB groups. By Day 10, the GP group exhibited significantly higher TGF-β levels than the GB group (*p* < 0.001), higher IL-10 levels than the GB and VE groups (*p* < 0.001), and lower IL-1β levels than the GB group (*p* < 0.001). By Day 18, IL-10 levels in the GP group were significantly higher than in the GB group (*p* < 0.001), while IL-1β and TNF-α levels were lower than in the GB and VE groups (*p* < 0.001). These findings indicate that GP, GB, and VE increased TGF-β and IL-10 levels while reducing IL-6, IL-1β, and TNF-α levels in UVB-damaged skin, with GP exhibiting superior effects to GB and comparable efficacy to VE.

### 3.5. Transcriptomic Profiling Reveals GP-Mediated Gene Regulation

To further investigate the molecular mechanism by which GP repairs UVB-induced skin damage, we analyzed the transcriptome of dorsal skin tissues. Correlation analysis of gene expression levels between samples (Figure S1A,B) showed that each group clustered separately, indicating reliable experimental results and appropriate sample selection. PCA (Figure S1C,D) revealed a clear separation between groups, suggesting significant differences among them.

To obtain a more detailed gene expression profile, we conducted differential gene expression analysis and determined the number of DEGs (Table 1, Figure S2). On Day 5, the largest number of DEGs compared to the MD group was observed in the CK group (5077 DEGs), followed by the VE group (1253), the GP group (1048), and the GB group (733). By Day 18, the number of DEGs between the GP and MD groups (734) exceeded that of the VE group (375). This indicates that the GB group exhibited the smallest gene expression differences compared to MD, whereas the GP group showed greater differences from MD than VE on Day 18. Compared to Day 5, the number of DEGs between MD and other groups decreased on Day 18, indicating that all groups exhibited some degree of recovery from UVB irradiation.

**Figure 6:**
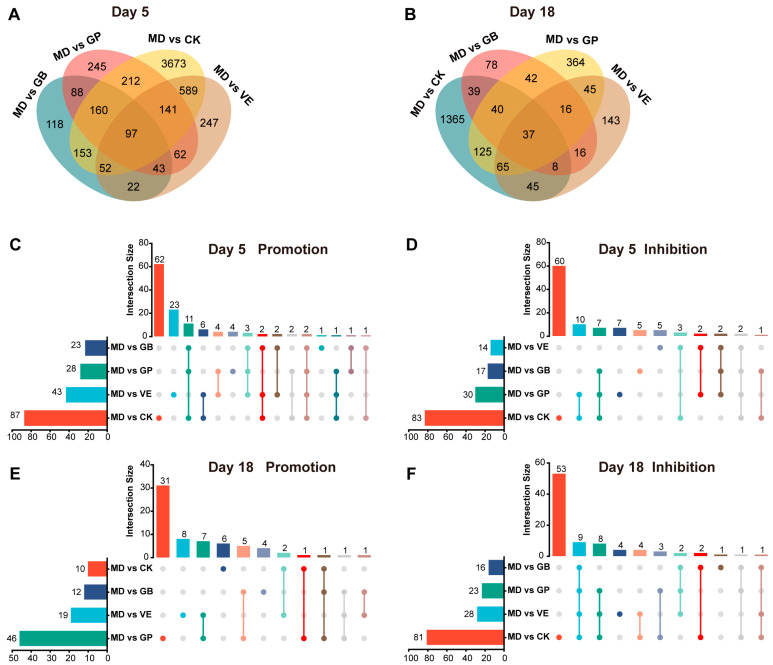
The transcriptome analysis in the dorsal skin of mice in each experimental group. (**A**,**B**) Venn diagram analysis of DEGs among groups on Day 5 and Day 18. (**C**–**F**) UpSet plot analysis of promoted and inhibited KEGG pathways among groups on Day 5 and Day 18.

Additionally, Venn diagram analysis of DEGs (Figure 6A,B) showed that on Day 5, the largest number of shared genes was found between MD vs. CK and MD vs. VE (879 genes), followed by MD vs. GP (610 genes), MD vs. GB (462 genes). Moreover, MD vs. GP shared more genes with MD vs. VE (343 genes) than with MD vs. GB (214 genes). On Day 18, the number of shared genes between MD vs. GP and MD vs. CK (267 genes) was greater than that between MD vs. VE (155 genes). These results suggest that, compared to the GB group, the GP group exhibited greater gene expression similarity to the CK and VE groups.

The top 10 DEGs were identified as key genes. On Day 5, the key genes in MD vs. GP were *Timp1*, *Col1a1*, *Aldh1a3*, *Sparc*, *Igfbp3*, *Aspn*, *Cxcl14*, *Thbs4*, *Galnt15*, and *Fbxo32*. Among them, eight genes were enriched in KEGG pathways, including ECM-receptor interaction, Focal adhesion, and PI3K-Akt signaling pathways, with one gene involved in the p53 signaling pathway and one gene in the FoxO signaling pathway (Table S4). On Day 18, the key genes in MD vs. GP were *Pvalb*, *Actn3*, *Casq1*, *Ryr1*, *Atp2a1*, *Acta1*, *Pygm*, *Pde4dip*, *Mybpc2*, and *Mylk4*, among which five genes were enriched in KEGG pathways, including the Calcium signaling pathway, cAMP signaling pathway, and cGMP-PKG signaling pathway, etc.

### 3.6. Effects of GP on KEGG Pathways in Mice with UVB-Induced Acute Photoaging Damage

#### 3.6.1. Analysis of Significantly Enriched Pathways

To investigate the cellular processes and functions associated with DEGs, KEGG enrichment analysis was separately performed on upregulated and downregulated DEGs, and significantly enriched pathways were screened (*p* < 0.05) (Table S5, Figures S3 and S4). On Day 5, compared to the MD group, the GP group significantly promoted 28 pathways and significantly inhibited 30 pathways. On Day 18, the GP group significantly promoted 46 pathways and inhibited 23 pathways. These pathways regulated oxidative stress, inflammation, and cellular damage in mice.

#### 3.6.2. Identification of Key Pathways

To explore the commonality and specificity of the significantly enriched pathways in each group, we performed an Upset plot analysis (Figure 6C–F). On Day 5, MD vs. GP shared 2 promotion pathways and 10 inhibitory pathways with MD vs. CK, 7 promotion pathways and 2 inhibitory pathways with VE, and 1 three-group shared promotion pathway (MD vs. GP/CK/VE). Meanwhile, the MD vs. GB group exhibited 1 promotion pathway and 1 inhibitory pathway shared with MD vs. CK, 2 promotion pathways shared with MD vs. VE, along with 2 three-group shared promotion pathways and 2 inhibitory pathways (MD vs. GB/CK/VE). On Day 18, MD vs. GP shared 1 promotion pathway and 3 inhibitory pathways with MD vs. CK, 7 promotion pathways with MD vs. VE, and 8 three-group shared inhibitory pathways (MD vs. GP/CK/VE). As for the MD vs. GB group, it exhibited 2 inhibitory pathways shared with MD vs. CK, 1 promotion pathway shared with MD vs. VE, and 1 three-group shared inhibitory pathway (MD vs. GB/CK/VE). These specific pathways are listed in Supplementary Table S6. Overall, compared to the MD vs. GB group, the MD vs. GP group shared more common pathways with the MD vs. CK and MD vs. VE groups. To gain deeper insights into the molecular mechanisms underlying the differential responses of GP and GB to UVB exposure, we designated MD vs. GP-specific pathways and the shared pathways among MD vs. GP, MD vs. CK, and MD vs. VE as key pathways (Table S6).

#### 3.6.3. Analysis of Key Pathways Network

**Figure 7:**
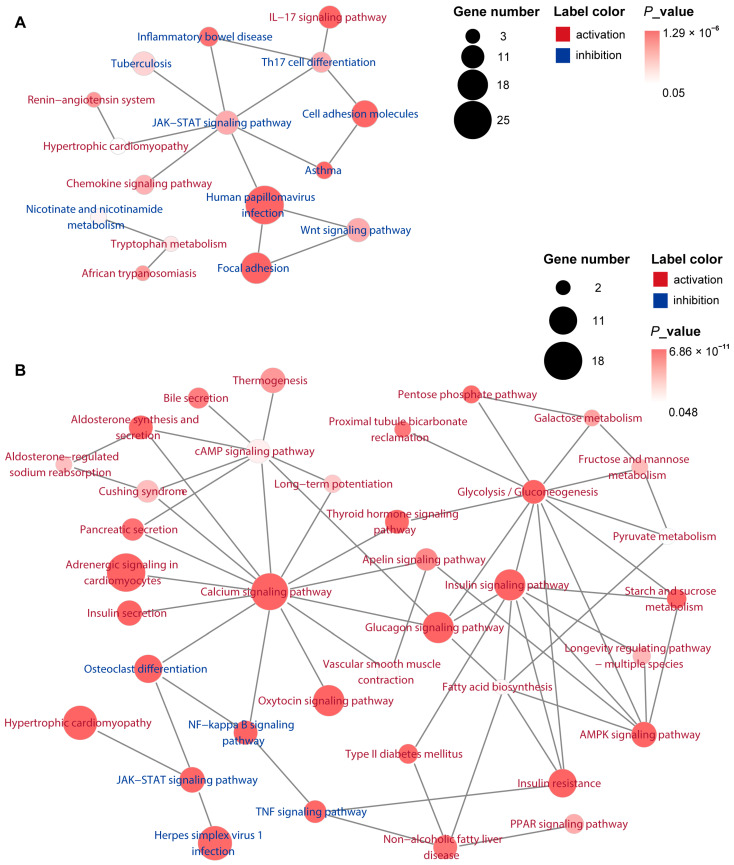
Enrichment network analysis of key KEGG pathways on Day 5 (**A**) and Day 18 (**B**).

To understand the interactions between key pathways and identify upstream and downstream signaling pathways, we conducted an enrichment network analysis. On Day 5, the key pathways were primarily centered around JAK-STAT signaling pathway, such as Wnt signaling pathway, Focal adhesion, IL-17 signaling pathway, and Th17 cell differentiation, etc. (Figure 7A). By Day 18, the majority of key pathways were promoted, with five pathways (including the TNF, NF-κB, and JAK-STAT signaling pathways) being inhibited. Key pathways formed a regulatory network centered around the Calcium signaling pathway, cAMP signaling pathway, Glycolysis/Gluconeogenesis, AMPK signaling pathway, and Insulin signaling pathway, etc. (Figure 7B).

## 4. Discussion

The global rise in photoaging and UVB-induced skin cancer underscores the urgent need for effective preventive and therapeutic strategies. Natural products with dual antioxidant and anti-inflammatory properties, such as polysaccharides [32,33,34], hold significant promise in dermatological applications. *G. elata* contains various bioactive components, with polysaccharides accounting for approximately 30% of its composition [35]. To better understand the mechanism by which GP alleviates UVB-induced acute skin damage, this study included GB as a control group. The results indicated that GP exhibited superior reparative effects on UVB-induced acute skin damage compared to GB and was comparable to the commonly used antioxidant VE.

GP consists of various monosaccharides linked by glycosidic bonds, forming linear or branched chains, with molecular weights reaching tens or even millions of Daltons [36]. Literature reports indicate that the molecular weight range of purified GP spans from 3.92 to 1913 kDa [37,38,39,40,41,42]. GP includes *Galactose* (*Gal*), *Glc*, *Xylose* (*Xyl*), *Mannose* (*Man*), and *Fru*, etc., with significant compositional differences among different *Gastrodia* varieties [28]. Variations in molecular weight and monosaccharide composition may be due to differences in *Gastrodia* species, geographical origin, and extraction methods [36]. FT-IR analysis confirmed the presence of O-H, C-H, C=O, and C-O bonds in GP, consistent with previous literature [21,43]. Acidic polysaccharides have been reported to exhibit strong antioxidant activity and protective effects against UVB irradiation [34]. Trace amounts of galacturonic acid in GP suggests weak acidity. However, the functional properties of polysaccharides are influenced by multiple factors, including molecular weight, monosaccharide composition, glycosidic linkages, and charge properties [17]. Due to experimental limitations, a definitive conclusion could not be drawn.

UVB-induced skin damage is characterized by increased epidermal thickness and loss of mature collagen fibers in the dermis [44]. In this study, mice exposed to UVB developed pigmentation, scaling, erythema, wrinkles, increased skin thickness, and traumatic scabbing, primarily due to the degradation of dermal elastic and collagen fibers following UVB exposure [7]. Our findings demonstrated that GP restored normal skin physiology by suppressing epidermal hyperplasia and collagen degradation. Over time, except for the CK group, all experimental groups exhibited improvement, likely reflecting the concurrent activation of intrinsic repair mechanisms in mice.

In oxidative stress states, UVB irradiation disrupted the balance between the oxidative and antioxidant defense systems of the skin. Hyperactivation of myeloperoxidase (MPO) exacerbates tissue injury by amplifying ROS generation [45]. ROS further oxidize polyunsaturated fatty acids in biomembranes, triggering lipid peroxidation [46,47] and producing malondialdehyde (MDA), a terminal product that disrupts cell membranes and induces cell death [20]. Besides SOD, GSH acts as a free radical scavenger and a cofactor for protective enzymes, mitigating oxidative cellular damage [48]. Our findings indicated that GP reduced ROS generation by inhibiting MPO activity, suppressed lipid peroxidation, and restored SOD and GSH enzymatic activity, thereby mitigating oxidative damage.

Reducing inflammation is essential for treating UVB-induced skin damage. Anti-inflammatory cytokines such as TGF-β and IL-10 are known to inhibit UVB-triggered cutaneous inflammation [49]. Notably, GP significantly attenuated pro-inflammatory cytokine production and normalized anti-inflammatory factor levels, confirming its capacity to suppress UVB-driven inflammation.

GP alleviated UVB-induced skin tissue damage by reducing ROS and eliminating inflammation, which was further confirmed by transcriptomic analysis. The transcriptomic results suggest that the repair process of GP for UVB-induced acute skin damage can be divided into two phases. In the early phase of repair (Day 5), GP primarily suppressed excessive inflammation and accelerated clearance of necrotic tissue and pathogens at the damage site through a key pathway network. The promotion of the IL-17 signaling pathway is well known to trigger inflammation [50]. However, in the early phase of repair, inflammation can accelerate the clearance of damaged cells and initiate the repair process [51]. The inhibition of the Th17 cell differentiation pathway reduces the secretion of the pro-inflammatory factor IL-17 and inhibits excessive inflammatory responses [52]. The inhibition of the JAK-STAT signaling pathway helps to suppress inflammation and regulate immune responses [21,53]. The promotion of the Wnt signaling pathway promotes the self-renewal and differentiation of hair follicle stem cells and epidermal stem cells [54], whereas its inhibition prevents excessive epidermal hyperplasia, reduces stem cell exhaustion, and maintains long-term barrier homeostasis. UVB irradiation damages the extracellular matrix (ECM) structure, and the promotion of Focal adhesion promotes cell migration and matrix regeneration [55], while its inhibition can prevent scarring due to excessive ECM deposition. On Day 5, key genes were mostly related to ECM.

In the late phase (Day 18), GP shifted focus to anti-inflammatory, antioxidant, and tissue repair processes. Promotion of the cAMP signaling pathway mitigates collagen degradation, skin aging, and melanin formation [56,57], while the promotion of the PPAR signaling pathway promotes stratum corneum lipid synthesis, accelerates barrier repair, reduces fibroblast overactivation, and lowers scarring risk [58,59,60]. The combined inhibition of the NF-κB signaling pathway, the TNF signaling pathway [61], and the JAK-STAT signaling pathway forms a five-target anti-inflammatory network that concurrently reduces inflammatory damage and enhances barrier repair. The promotion of the AMPK signaling pathway can clear damaged molecules through autophagy, maintaining genomic stability [62,63,64]. The promotion of the Glycolysis/Gluconeogenesis [65] and the Insulin signaling pathway [66,67] can provide energy and synthetic materials for skin repair, enhancing collagen synthesis by fibroblasts. The promotion of the Calcium signaling pathway [68,69] regulates immune cell migration, optimizing the inflammation resolution and the repair process. Notably, many of the key genes upregulated on Day 18 were involved in the Calcium signaling pathway. In summary, UVB-induced skin damage repair is a highly coordinated process that involves multiple mechanisms, including energy metabolism, inflammation regulation, and cell proliferation and migration. In the later stage of repair, GP exhibited an efficient, low-inflammation, and energy-sufficient repair network.

Currently, only one published study has explored the application of GP in UVB-induced skin damage [35]. This prior work primarily described the preparation and evaluation of gastrodin microsphere-loaded *G. elata* polysaccharide composite hydrogel on UVB-induced skin damage. Our study differs in three critical aspects: (1) Whereas the reported composite hydrogel comprised oxidized *G. elata* polysaccharide, carboxymethyl chitosan, and gastrodin microspheres, we focused exclusively on the therapeutic potential of unmodified GP as a single compound; (2) While the aforementioned study demonstrated the hydrogel’s protection via suppression of pro-inflammatory cytokines and modulation of apoptosis-related pathways, we comprehensively delineated GP’s reparative mechanisms through integrated analysis of its antioxidant/anti-inflammatory activities and dynamic pathway network interactions across treatment phases; (3) The prior study focused on the preparation of composite hydrogels, representing an effective strategy for the practical application of *G. elata* polysaccharide. In contrast, our research specifically investigates the effects and mechanisms of GP in repairing UVB-induced acute skin damage. Together, these two studies provide important insights for the application of the natural product GP in dermatological medicine. Nevertheless, research on GP’s repair of UVB-induced acute skin damage warrants further investigation. For instance, purifying the GP and conducting more detailed characterization of its structural features could help elucidate the specific bioactive components responsible for its effects. Furthermore, although our study identified potential signaling pathways involved in GP-mediated repair of UVB-induced skin damage, the proposed mechanisms remain preliminary; key nodes and interactions within the identified networks require additional functional validation to confirm their roles and establish definitive therapeutic targets.

## 5. Conclusions

**Figure 8:**
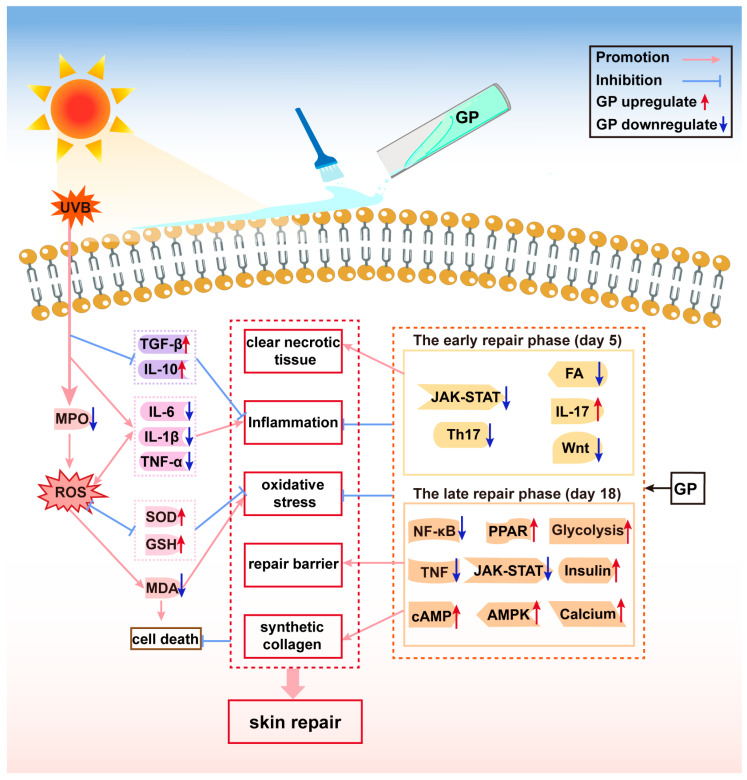
Potential mechanism of GP in repairing the UVB-induced acute skin damage.

In conclusion, our findings suggest that GP mitigates UVB-induced acute skin damage through a series of antioxidative, anti-inflammatory, energy metabolism, cell migration, and proliferation mechanisms. These mechanisms are coordinated through key pathway networks, including the JAK-STAT signaling pathways, the calcium signaling pathways, and so on (Figure 8). This protective mechanism operates through three synergistic axes: (1) GP restores SOD/GSH expression, suppresses excessive ROS production, and alleviates oxidative damage; (2) GP downregulates pro-inflammatory cytokines (TNF-α, IL-1β, IL-6) while restoring anti-inflammatory mediators (TGF-β, IL-10), effectively alleviating skin inflammation; (3) GP facilitates the clearance of necrotic tissue, promotes collagen synthesis, and accelerates skin barrier repair. By orchestrating these responses, GP achieves comprehensive repair of UVB-induced acute skin damage, demonstrating its potential as a multi-target therapeutic agent and offering novel strategies for developing safe and effective skin protectants.

## Figures and Tables

**Table 1:** Number of DEGs between groups.

Time	Control vs. Treatment	Upregulation	Downregulation	Total
Day 5	MD vs. GP	320	728	1048
	MD vs. GB	380	353	733
	MD vs. VE	276	977	1253
	MD vs. CK	1885	3192	5077
Day 18	MD vs. GP	433	301	734
	MD vs. GB	98	178	276
	MD vs. VE	190	185	375
	MD vs. CK	257	1467	1724

## Data Availability

The transcriptome sequences generated in this study have been submitted to GenBank with the accession number is PRJNA1250770. The bioproject is PRJNA1250770. (https://dataview.ncbi.nlm.nih.gov/object/PRJNA1250770) (accessed on 14 July 2025).

## Supplementary Materials

The following supporting information can be downloaded at: https://www.mdpi.com/article/10.3390/antiox14070894/s1, Figure S1: Correlation analysis of gene expression levels between samples (A); Principal component analysis (PCA) for the correlation among samples in each group (B). Figure S2: Volcano plots of DEGs between the MD group and other groups. (A-D) Volcano plots of DEGs on Day 5. (E-H) Volcano plots of DEGs on Day 18. Figure S3: Significantly enriched pathways of upregulated DEGs on Day 5 (A–C) and Day 18 (D–F). Figure S4: Significantly enriched pathways of downregulated DEGs on Day 5 (A–C) and Day 18 (D–F). Table S1 DAI coring criteria. Table S2: *p*-value for oxidative stress biomarkers. Table S3: *p*-values for the expression levels of pro/anti-inflammatory cytokines. Table S4: The key genes in MD vs. GP. Table S5: The pathways significantly enriched in the MD vs. CK group. Table S6: Common and unique pathways of each group and key pathways.
