# Supplementary material for: Antioxidant and Anti-Inflammatory Effects of Crude Gastrodia elata Polysaccharides in UVB-Induced Acute Skin Damage
Source: Antioxidants (Basel). 2025 Jul 21;14(7):894. doi: 10.3390/antiox14070894 (PMC12292105; doi:10.3390/antiox14070894)
Supplement: Supplementary file 1 [file antioxidants-14-00894-s001.zip › Supplementary material.pdf]

Supplementary Material

Supplementary figures:

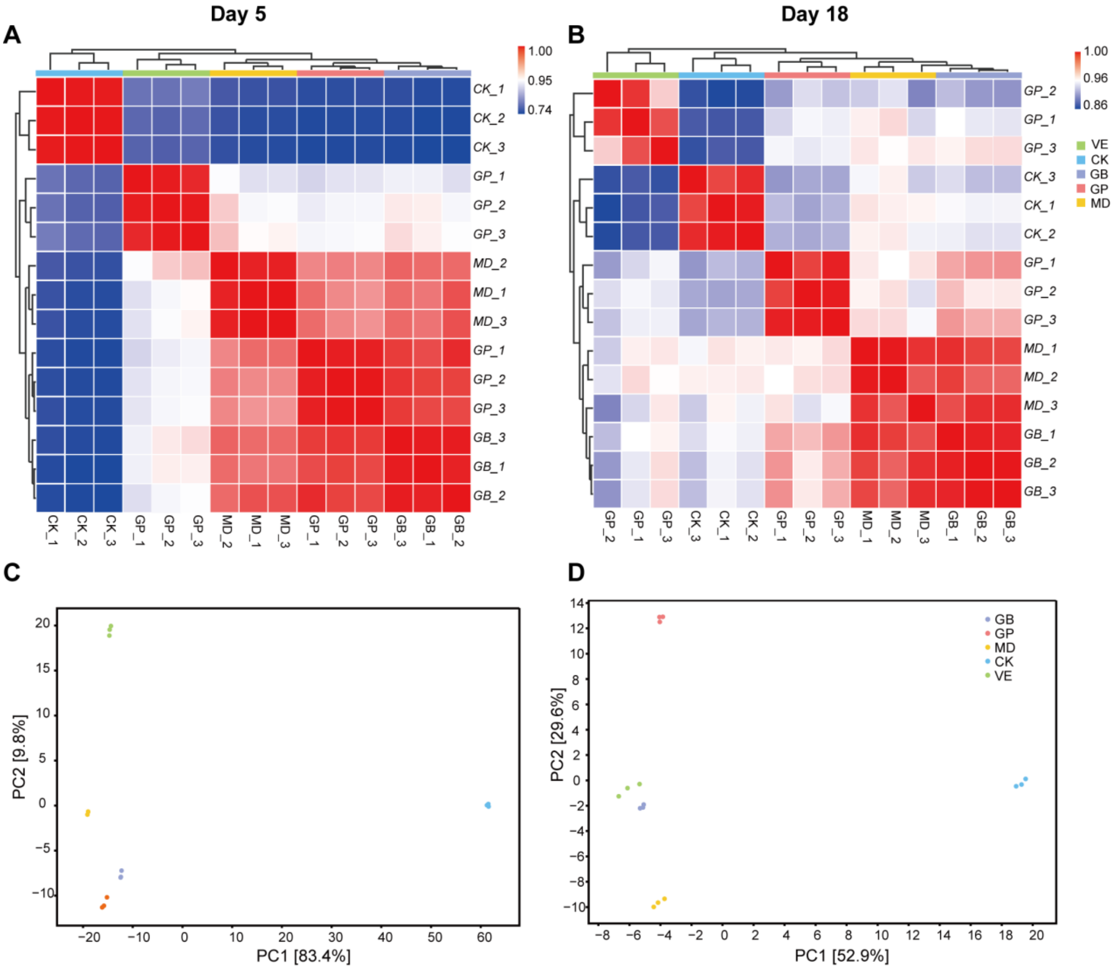

Figure S1. Correlation analysis of gene expression levels between samples (A); Principal component analysis (PCA) for the correlation among samples in each group (B).

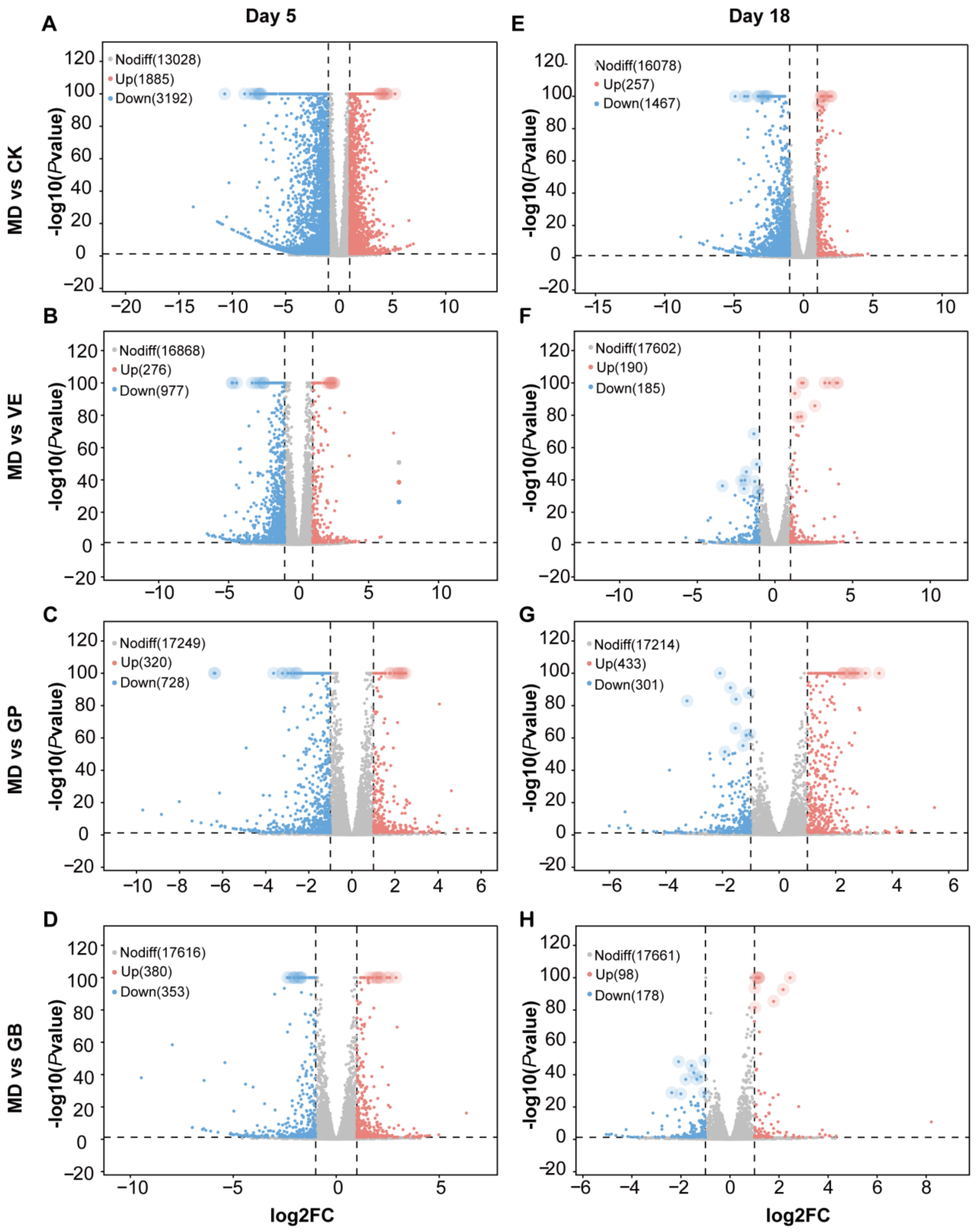

Figure S2. Volcano plots of DEGs between the MD group and other groups. (A-D) Volcano plots of DEGs on Day 5. (E-H) Volcano plots of DEGs on Day 18.

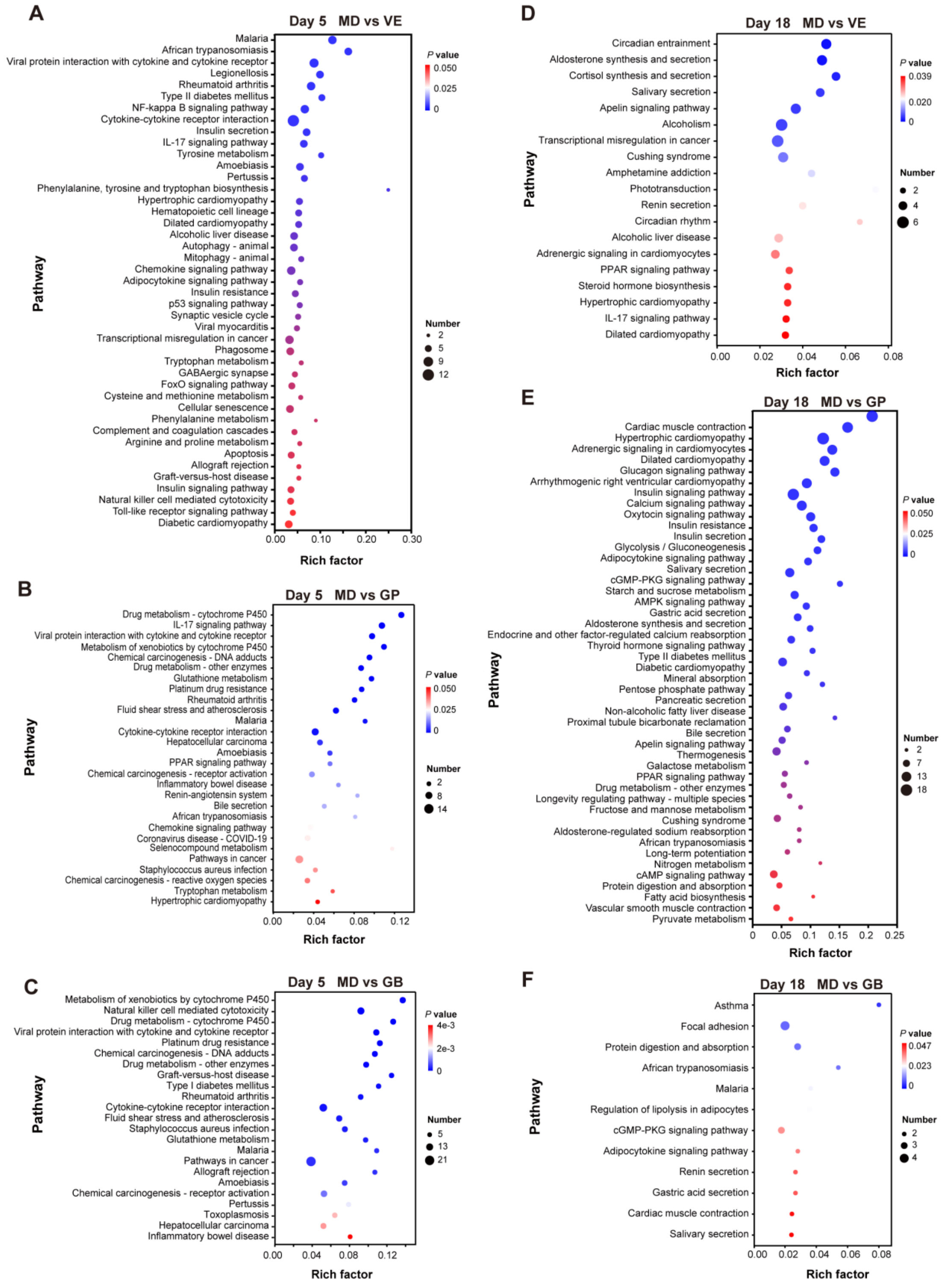

Figure S3. Significantly enriched pathways of upregulated DEGs on Day 5 (A-C) and Day 18 (D-F).

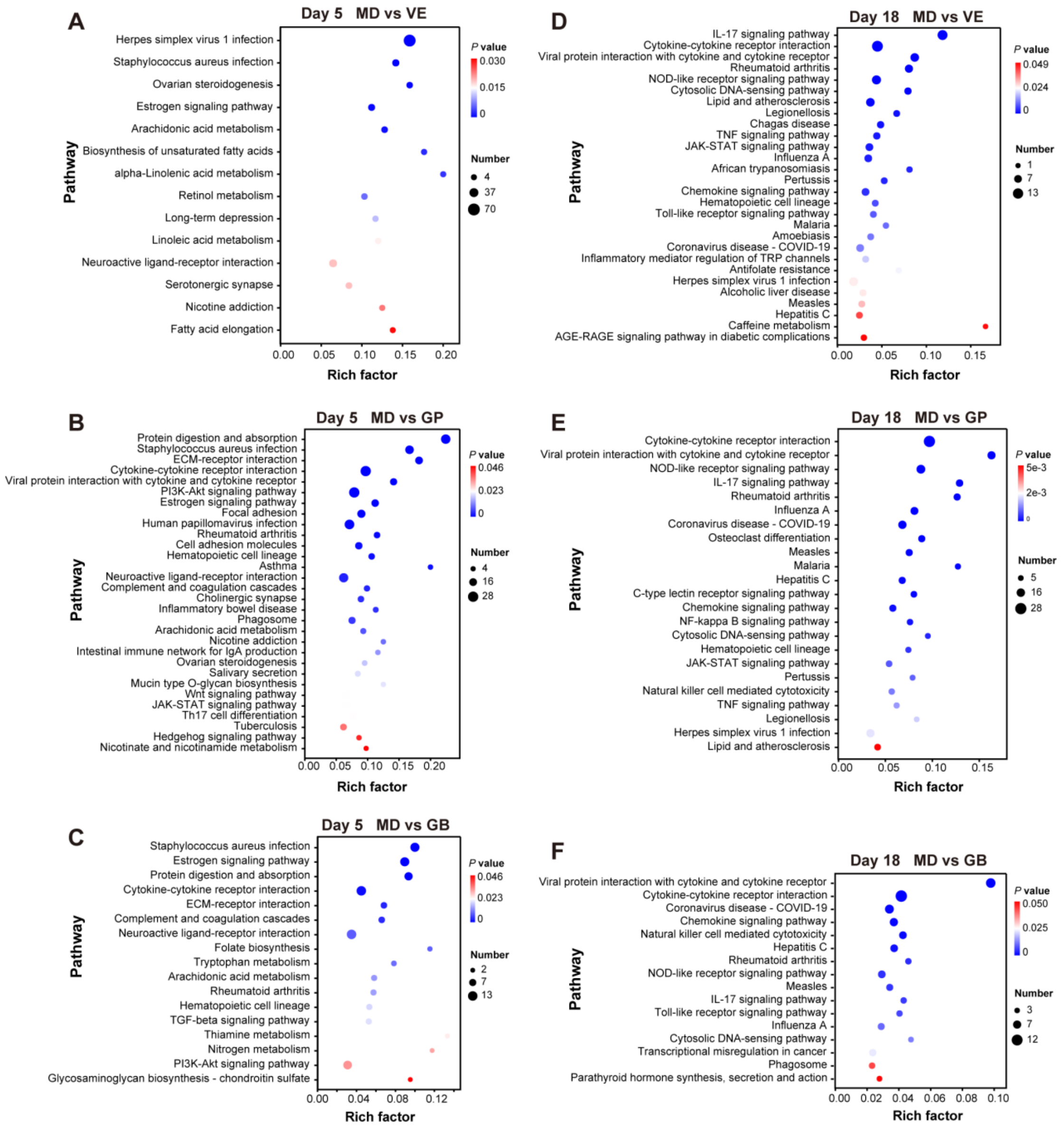

Figure S4. Significantly enriched pathways of downregulated DEGs on Day 5 (A-C) and Day 18 (D-F).

Supplementary tables:

Table S1. DAI coring criteria

Index	0 points	1 points	2 points	3 points
Pigmentation	None	Light brown	Medium brown	Dark brown
Scaling	None	Slight	Moderate	Significant
Erythema	None	Mild	Moderate	With edema, crust, and necrosis
Wrinkles	None	Mild	Moderate	Wrinkles
Elasticity	Good	Fair	Poor	Complete loss
Skin thickness	Normal	Mild thickening	Moderate thickening	Marked thickening

Table S2. *P*-value for oxidative stress biomarkers

Time	Group	SOD	GSH	MPO	MDA
Day5	MD vs CK	< 0.001	< 0.001	< 0.001	< 0.001
	VE vs CK	< 0.001	< 0.001	< 0.001	< 0.001
	VE vs MD	/	< 0.001	< 0.001	< 0.001
	GP vs CK	< 0.001	< 0.001	< 0.001	< 0.001
	GP vs MD	< 0.001	< 0.001	< 0.001	< 0.001
	GP vs VE	/	/	/	/
	GB vs CK	< 0.001	< 0.001	< 0.001	< 0.001
	GB vs MD	< 0.01	< 0.001	< 0.001	< 0.001
	GB vs VE	/	< 0.001	/	< 0.01
	GB vs GP	/	< 0.001	/	/
Day10	MD vs CK	< 0.001	< 0.001	< 0.001	< 0.001
	VE vs CK	/	< 0.001	< 0.001	< 0.001
	VE vs MD	< 0.001	< 0.001	< 0.001	< 0.001
	GP vs CK	/	< 0.01	< 0.001	< 0.001
	GP vs MD	< 0.001	< 0.001	< 0.001	< 0.001
	GP vs VE	/	/	/	/
	GB vs CK	< 0.01	< 0.001	< 0.001	< 0.001
	GB vs MD	< 0.001	< 0.001	< 0.01	< 0.001
	GB vs VE	/	< 0.01	/	/
	GB vs GP	/	< 0.001	< 0.001	/
Day18	MD vs CK	< 0.01	< 0.001	< 0.001	< 0.001
	VE vs CK	/	/	= 0.024	/
	VE vs MD	= 0.014	< 0.001	< 0.001	< 0.001
	GP vs CK	/	< 0.001	= 0.045	/
	GP vs MD	= 0.024	< 0.001	< 0.001	< 0.001
	GP vs VE	/	< 0.001	/	/
	GB vs CK	/	< 0.001	< 0.001	< 0.01
	GB vs MD	/	< 0.001	< 0.001	< 0.001
	GB vs VE	/	< 0.001	/	= 0.015
	GB vs GP	/	/	/	< 0.01

Table S3. *P*-values for the expression levels of pro/anti-inflammatory cytokines

Time	Group	TGF-β	1L-10	1L-6	1L-β	TNF-α
Day5	MD vs CK	< 0.001	< 0.001	< 0.001	< 0.001	< 0.001
	VE vs CK	< 0.001	< 0.001	< 0.001	< 0.001	< 0.001
	VE vs MD	= 0.017	< 0.001	= 0.041	< 0.001	< 0.001
	GP vs CK	< 0.001	< 0.001	< 0.001	< 0.001	< 0.001
	GP vs MD	< 0.001	< 0.001	< 0.001	< 0.001	< 0.001
	GP vs VE	< 0.001	< 0.001	< 0.001	< 0.001	< 0.01
	GB vs CK	< 0.001	< 0.001	< 0.001	< 0.001	< 0.001
	GB vs MD	< 0.001	< 0.001	< 0.001	< 0.001	< 0.001
	GB vs VE	< 0.01	< 0.001	< 0.001	< 0.001	/
	GB vs GP	/	/	/	< 0.001	< 0.001
Day10	MD vs CK	< 0.001	< 0.001	< 0.001	< 0.001	< 0.001
	VE vs CK	/	< 0.001	< 0.001	< 0.001	< 0.001
	VE vs MD	< 0.001	< 0.001	< 0.001	< 0.001	< 0.001
	GP vs CK	/	/	< 0.01	< 0.001	< 0.001
	GP vs MD	< 0.001	< 0.001	< 0.001	< 0.001	< 0.001
	GP vs VE	/	< 0.001	< 0.001	/	< 0.001
	GB vs CK	< 0.001	< 0.001	< 0.001	< 0.001	< 0.001
	GB vs MD	< 0.001	< 0.001	< 0.001	< 0.001	< 0.001
	GB vs VE	< 0.001	/	< 0.001	< 0.001	/
	GB vs GP	< 0.001	< 0.001	/	< 0.001	/
Day18	MD vs CK	< 0.001	/	< 0.001	< 0.001	< 0.001
	VE vs CK	/	< 0.001	< 0.001	< 0.001	/
	VE vs MD	< 0.001	< 0.001	< 0.001	< 0.001	< 0.001
	GP vs CK	/	< 0.001	< 0.001	< 0.01	= 0.030
	GP vs MD	< 0.001	< 0.001	< 0.001	< 0.001	< 0.001
	GP vs VE	/	/	< 0.001	< 0.001	/
	GB vs CK	/	/	< 0.001	< 0.001	< 0.001
	GB vs MD	< 0.001	< 0.001	< 0.001	< 0.001	= 0.021
	GB vs VE	< 0.01	< 0.001	< 0.001	< 0.001	< 0.001
	GB vs GP	/	< 0.001	= 0.011	< 0.001	< 0.001
