# Supplementary figures and images for: Antioxidant and Anti-Inflammatory Effects of Crude Gastrodia elata Polysaccharides in UVB-Induced Acute Skin Damage
Source: Antioxidants (Basel). 2025 Jul 21;14(7):894. doi: 10.3390/antiox14070894 (PMC12292105; doi:10.3390/antiox14070894)

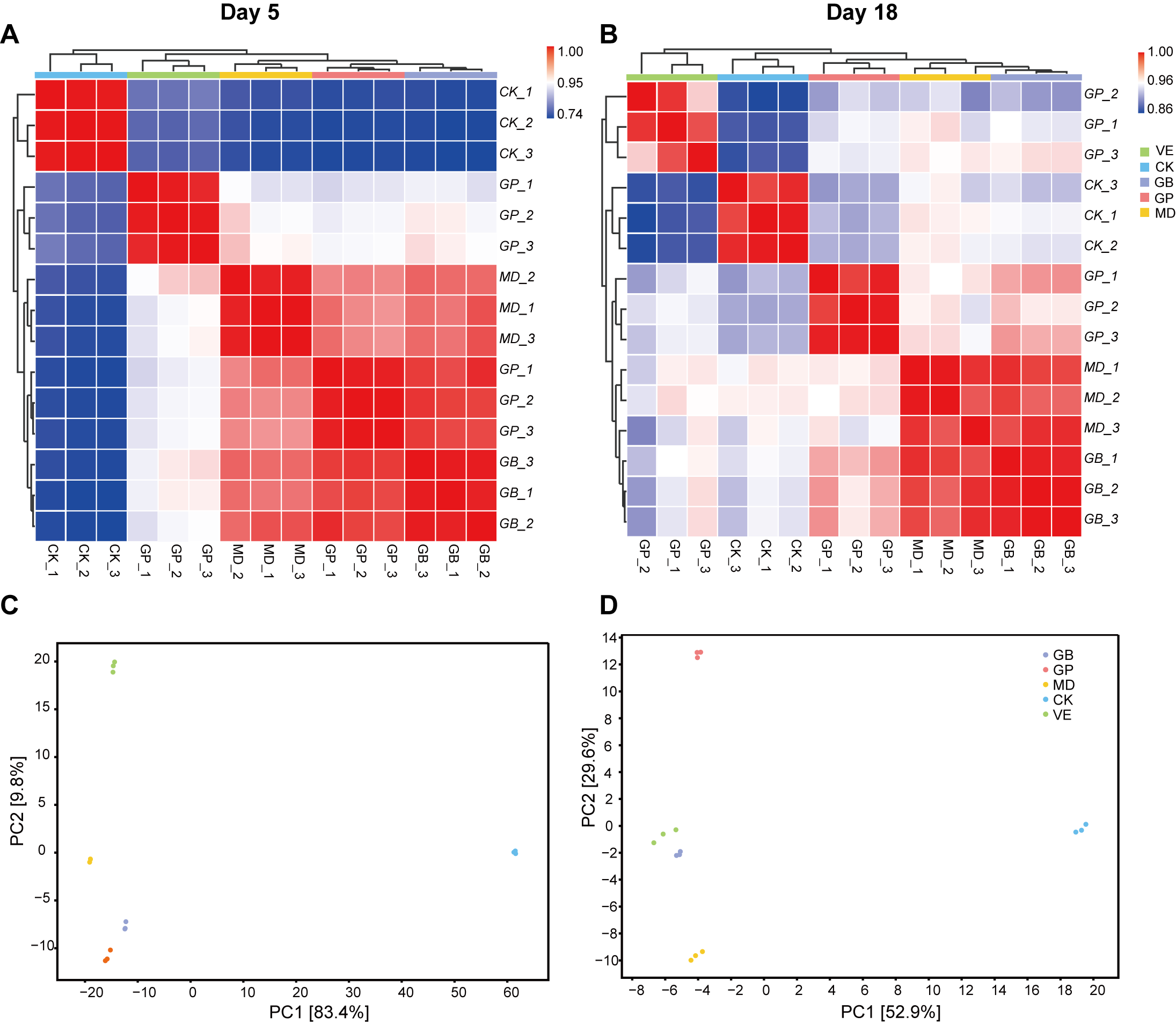

Supplement: Supplementary file 1 [file antioxidants-14-00894-s001.zip › Figure S1.tif]

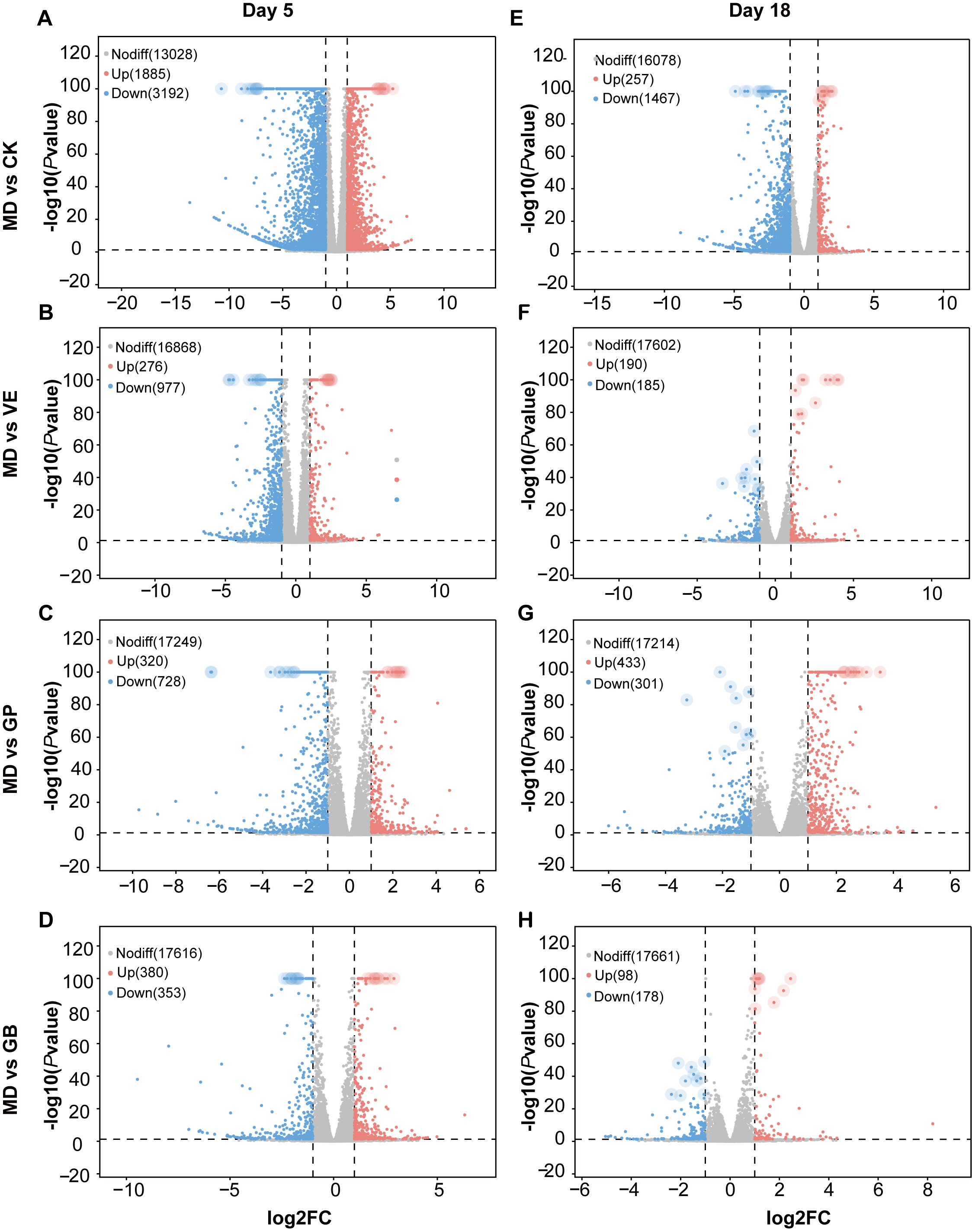

Supplement: Supplementary file 1 [file antioxidants-14-00894-s001.zip › Figure S2.tif]

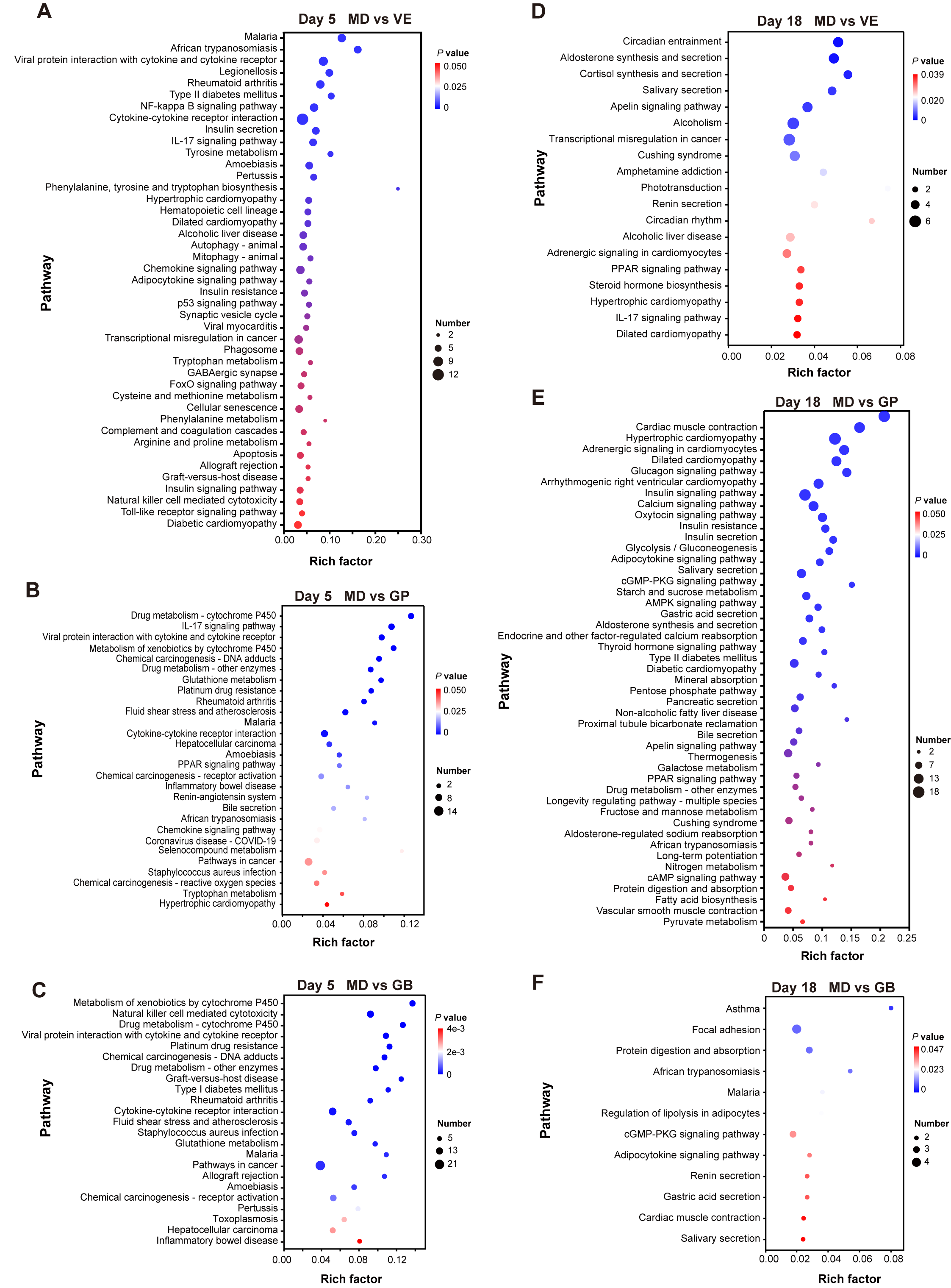

Supplement: Supplementary file 1 [file antioxidants-14-00894-s001.zip › Figure S3.tif]

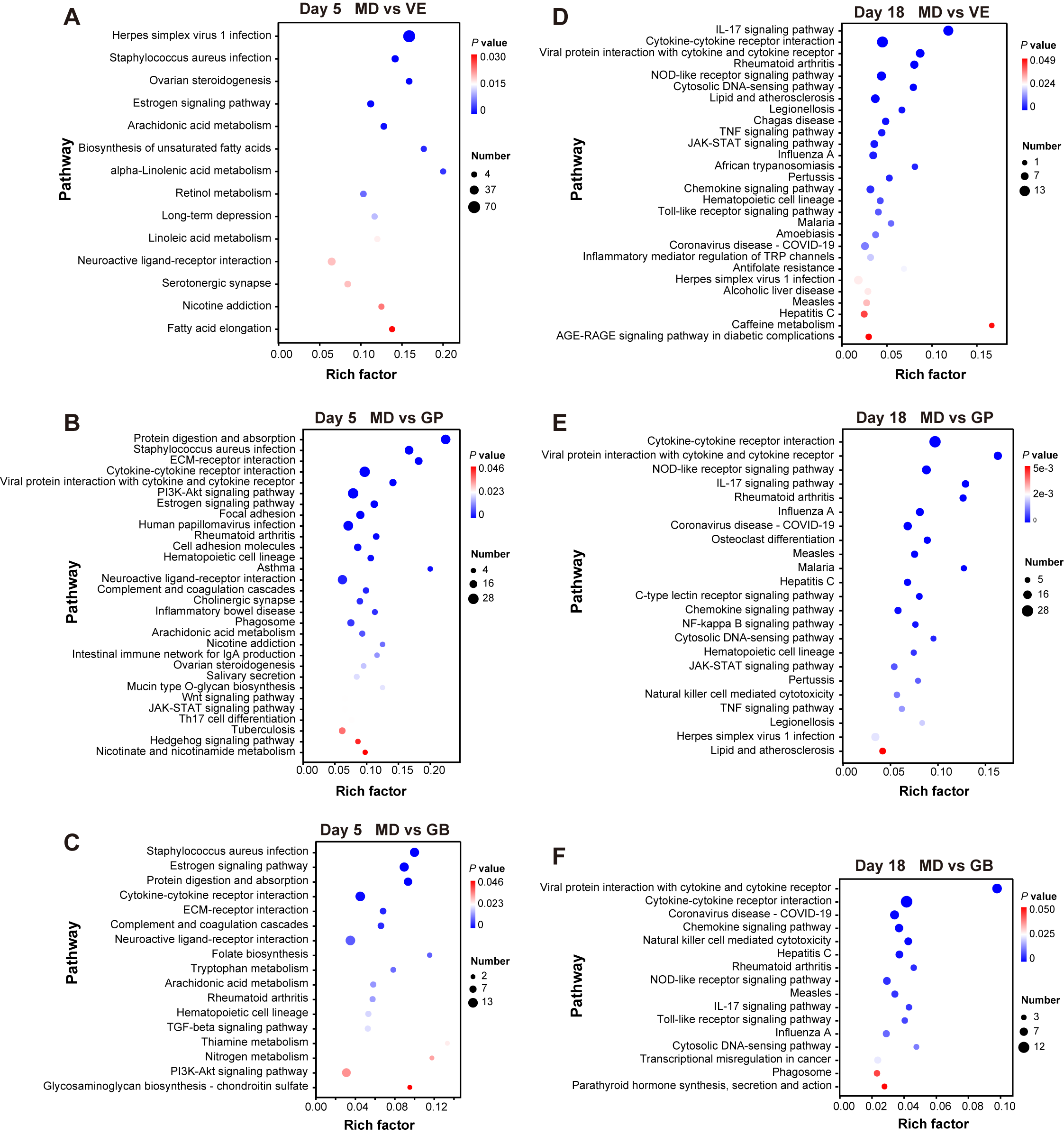

Supplement: Supplementary file 1 [file antioxidants-14-00894-s001.zip › Figure S4.tif]
